# Procedural outcomes in patients with dual versus single antiplatelet therapy prior to transcatheter aortic valve replacement

**DOI:** 10.1038/s41598-021-94599-2

**Published:** 2021-07-29

**Authors:** Hatim Seoudy, Maren Thomann, Johanne Frank, Matthias Lutz, Thomas Puehler, Georg Lutter, Oliver J. Müller, Norbert Frey, Mohammed Saad, Derk Frank

**Affiliations:** 1 Department of Internal Medicine III, Cardiology and Angiology, University Hospital Schleswig-Holstein, Campus Kiel, Arnold-Heller-Str. 3, 24105 Kiel, Germany; 2 DZHK (German Centre for Cardiovascular Research), Partner Site Hamburg, Kiel, Lübeck, Germany; 3 Department of Cardiac and Vascular Surgery, University Hospital Schleswig-Holstein, Campus Kiel, Kiel, Germany; 4 Department of Cardiology, Angiology and Pneumology, Internal Medicine III, Medical Hospital, Heidelberg University Hospital, Heidelberg, Germany; 5 DZHK (German Centre for Cardiovascular Research), Partner Site Heidelberg/Mannheim, Heidelberg, Germany

**Keywords:** Cardiac device therapy, Interventional cardiology

## Abstract

The impact of uninterrupted dual antiplatelet therapy (DAPT) on bleeding events among patients undergoing transcatheter aortic valve replacement (TAVR) has not been well studied. We conducted an analysis of 529 patients who underwent transfemoral TAVR in our centre and were receiving either DAPT or single antiplatelet therapy (SAPT) prior to the procedure. Accordingly, patients were grouped into a DAPT or SAPT group. Following current guidelines, patients in the SAPT group were switched to DAPT for 90 days after the procedure. The primary endpoint of our analysis was the incidence of bleeding events at 30 days according to the VARC-2 classification system. Any VARC-2 bleeding complications were found in 153 patients (28.9%), while major/life-threatening or disabling bleeding events occurred in 60 patients (11.3%). Our study revealed no significant difference between the DAPT vs. SAPT group regarding periprocedural bleeding complications. Based on multivariable analyses, major bleeding (HR 4.59, 95% CI 1.64–12.83, p = 0.004) and life-threatening/disabling bleeding (HR 8.66, 95% CI 3.31–22.65, p < 0.001) events were significantly associated with mortality at 90 days after TAVR. Both pre-existing DAPT and SAPT showed a comparable safety profile regarding periprocedural bleeding complications and mortality at 90 days. Thus, DAPT can be safely continued in patients undergoing transfemoral TAVR.

## Introduction

Dual antiplatelet therapy (DAPT), consisting of aspirin and a P2Y12 receptor inhibitor, constitutes an essential treatment strategy after percutaneous coronary intervention (PCI) and myocardial infarction (MI)^1,2^. As DAPT may be associated with periprocedural bleeding in non-cardiac and cardiac interventions, it is interrupted and switched to single antiplatelet therapy (SAPT) in certain scenarios. In contrast, discontinuation of DAPT potentially exposes patients to adverse ischemic events^3,4^. Management of DAPT therefore requires careful consideration of both bleeding and ischemic risks^5^.

Transcatheter aortic valve replacement (TAVR) has emerged as an essential treatment option for patients with symptomatic aortic stenosis (AS)^6,7^. As coronary artery disease and AS share common risk factors, a significant number of AS patients have an appropriate indication for preprocedural DAPT or SAPT^8^. In the context of TAVR, recently published and current studies mainly focus on the optimal antiplatelet and antithrombotic regimen after the procedure^6,9^. However, the impact of uninterrupted DAPT on adverse outcomes compared to SAPT in patients undergoing TAVR has not been well defined. The main objective of our study was to evaluate whether continuation of DAPT was associated with increased periprocedural complications in TAVR recipients.

## Methods

### Study design

**Figure 1:**
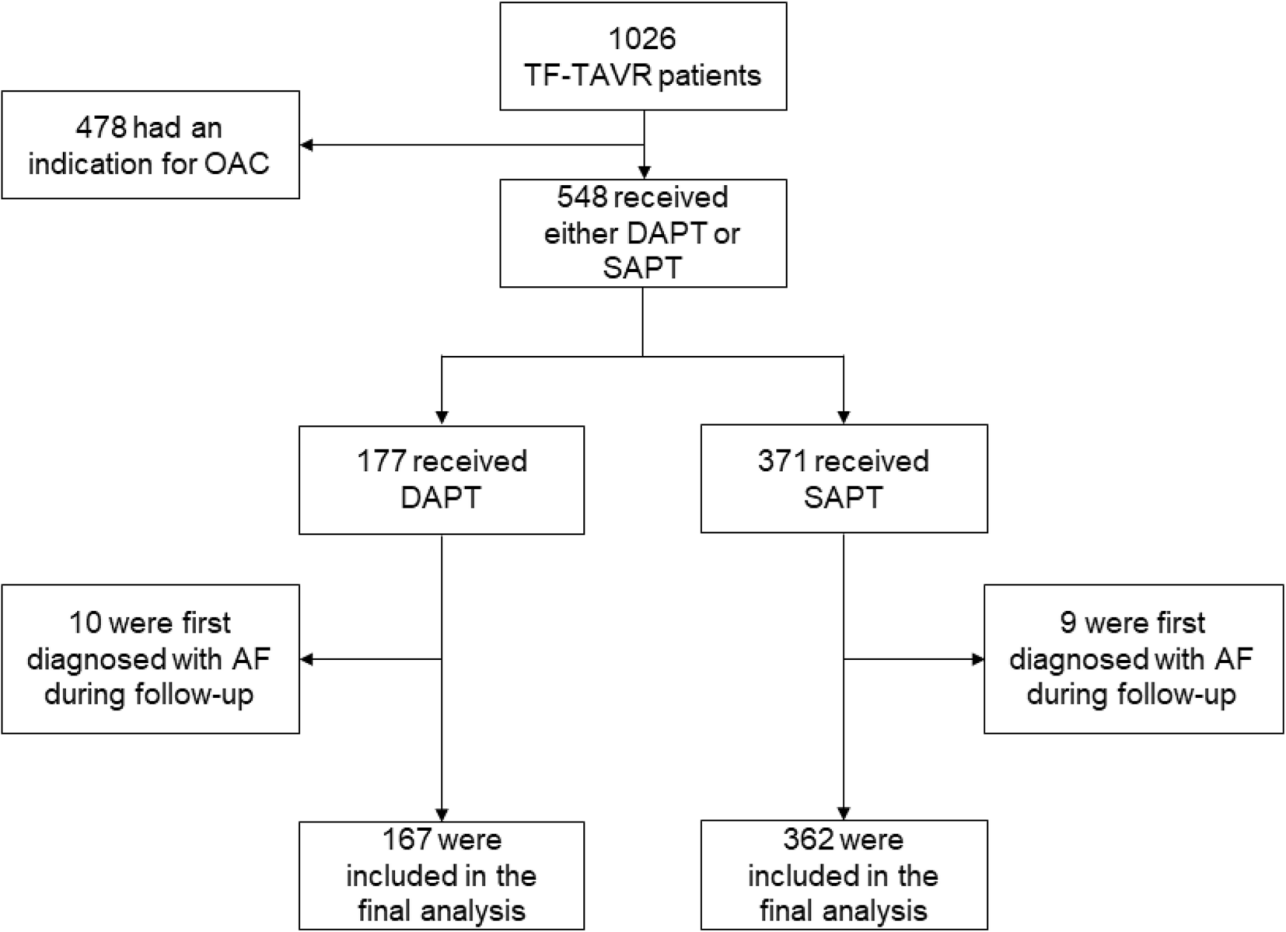
Study design. A total of 1026 patients undergoing transfemoral TAVR had been prospectively enrolled. During follow-up, 19 patients were excluded due to the diagnosis of atrial fibrillation. Finally, the DAPT group comprised 167 patients and the SAPT group 362 patients.

Between February 2014 and May 2020, a total of 1026 patients undergoing transfemoral TAVR using a third-generation device at the University Hospital Schleswig–Holstein, Kiel, Germany, had been prospectively enrolled in an observational TAVR database (Fig. 1). Of these, 548 patients received either DAPT or SAPT prior to the procedure and were therefore selected for our study. Patients were grouped into a DAPT or SAPT group, accordingly. Based on current ESC/EACTS guidelines, all patients with pre-existing SAPT were switched to DAPT (consisting of aspirin and clopidogrel) on the first day after the procedure for a total of 90 days^6^. During follow-up, 19 patients were excluded from our study, as they were first diagnosed with atrial fibrillation and switched to oral anticoagulation therapy. The primary endpoint was the incidence of periprocedural bleeding complications at 30 days in accordance with the definitions of the Valve Academic Research Consortium-2 (VARC-2) system which uses Bleeding Academic Research Consortium (BARC) classifications^10^.

### Procedural details

All TAVR procedures were performed using either the SAPIEN devices (Edwards Lifesciences, Irvine, California) or the CoreValve Evolut R/PRO devices (Medtronic, Minneapolis, Minnesota). Optimal type and size of transcatheter heart valve were determined using preprocedural multidetector CT measurements evaluated with 3mensio Structural Heart software (3mensio Medical Imaging BV, Bilthoven, The Netherlands). Pre-dilatation and post-dilatation were left to the physician’s discretion. During the TAVR procedure, unfractionated heparin was administered to achieve an activated clotting time of 250–300 s. Closure of the vascular access was usually performed using two Perclose ProGlide™ vascular closure systems (Abbott Laboratories, Chicago, IL, USA).

### Data collection

Written informed consent was obtained from each patient. The study was approved by the Ethics Committee at the University of Kiel and conformed to the ethical guidelines of the Declaration of Helsinki^11^. Patient data and blood samples were collected 1(–3) days prior to TAVR. Follow-up after discharge included a visit in our cardiology outpatient clinic 1–3 months after TAVR, as well as an annual phone call follow-up.

### Bleeding complications

Bleeding events at 30 days were systematically evaluated following the standardized VARC-2 definitions^10^. Even in the absence of clinical overt bleeding, the majority of patients (86.2%) underwent postprocedural assessment using color flow Doppler ultrasound as part of our TAVR fast-track programme. Within the DAPT group, we also analysed the association of the PRECISE-DAPT score with periprocedural bleeding events at 30 days^12^.

### Life-threatening bleeding

Definition: Fatal bleeding (BARC type 5) OR bleeding in a critical organ, such as intracranial, intraspinal, intraocular, or pericardial necessitating pericardiocentesis, or intramuscular with compartment syndrome (BARC type 3b and 3c) OR bleeding causing hypovolaemic shock or severe hypotension requiring vasopressors or surgery (BARC type 3b) OR overt source of bleeding with drop in haemoglobin ≥ 5 g/dl or whole blood or packed red blood cells (RBCs) transfusion ≥ 4 units (BARC type 3b).

### Major bleeding (BARC type 3a)

Definition: Overt bleeding either associated with a drop in the haemoglobin level of at least 3.0 g/dl or requiring transfusion of two or three units of whole blood/RBC, or causing hospitalization or permanent injury, or requiring surgery AND does not meet criteria of life-threatening or disabling bleeding.

### Minor bleeding (BARC type 2 or 3a, depending on the severity)

Definition: Any bleeding worthy of clinical mention (e.g. access site haematoma) that does not qualify as life-threatening, disabling, or major.

### Statistical analyses

Continuous data are presented as median and interquartile range (IQR) or mean (± SD), as applicable, based on the Shapiro–Wilk test for normality. Categorical data are summarized as frequencies (percentage, %). Data were analysed using the Mann–Whitney-U and Student’s test, as applicable, as well as the χ^2^-test; in case of few observations (frequency less than 10 for an individual cell), Fisher’s exact test was used. Mortality at 90 days was assessed using the log-rank test. Preprocedural variables significantly associated with mortality at 90 days were included in a Cox regression model. Backward selection was based on the likelihood ratio criteria. For each variable, the proportional hazards assumption was approved by testing for interactions between Schoenfeld residuals and the log‐transformed time using the function “cox.zph()” of the “R [survival] package”. Results are summarized as adjusted hazard ratios (HRs) with 95% confidence intervals (CI). The diagnostic ability of the PRECISDE-DAPT score for stratifying bleeding risk was determined using receiver operating characteristic (ROC) curve analysis. A two-sided p-value of < 0.05 was considered statistically significant. All statistical analyses were performed using the statistical software R, version 4.0.4, and GraphPad PRISM, version 8.

### Ethics

All participants gave informed consent. This study was conducted in accordance with the Declaration of Helsinki and was approved by the local ethics committee at the University of Kiel.

## Results

A total of 548 TAVR patients with a complete dataset were available for analysis using our institution’s TAVR database (University Hospital Schleswig–Holstein Kiel, Germany). Based on preprocedural antiplatelet therapy, patients were assigned either to the DAPT (n = 177) or SAPT (n = 371) group. During follow-up, 10 patients in the DAPT group and 9 in the SAPT group were first diagnosed with atrial fibrillation. These patients were switched to oral anticoagulation therapy and were thus excluded from our study. For our final analysis, the DAPT group comprised 167 patients and the SAPT group 362 patients (Fig. 1).

**Figure 2:**
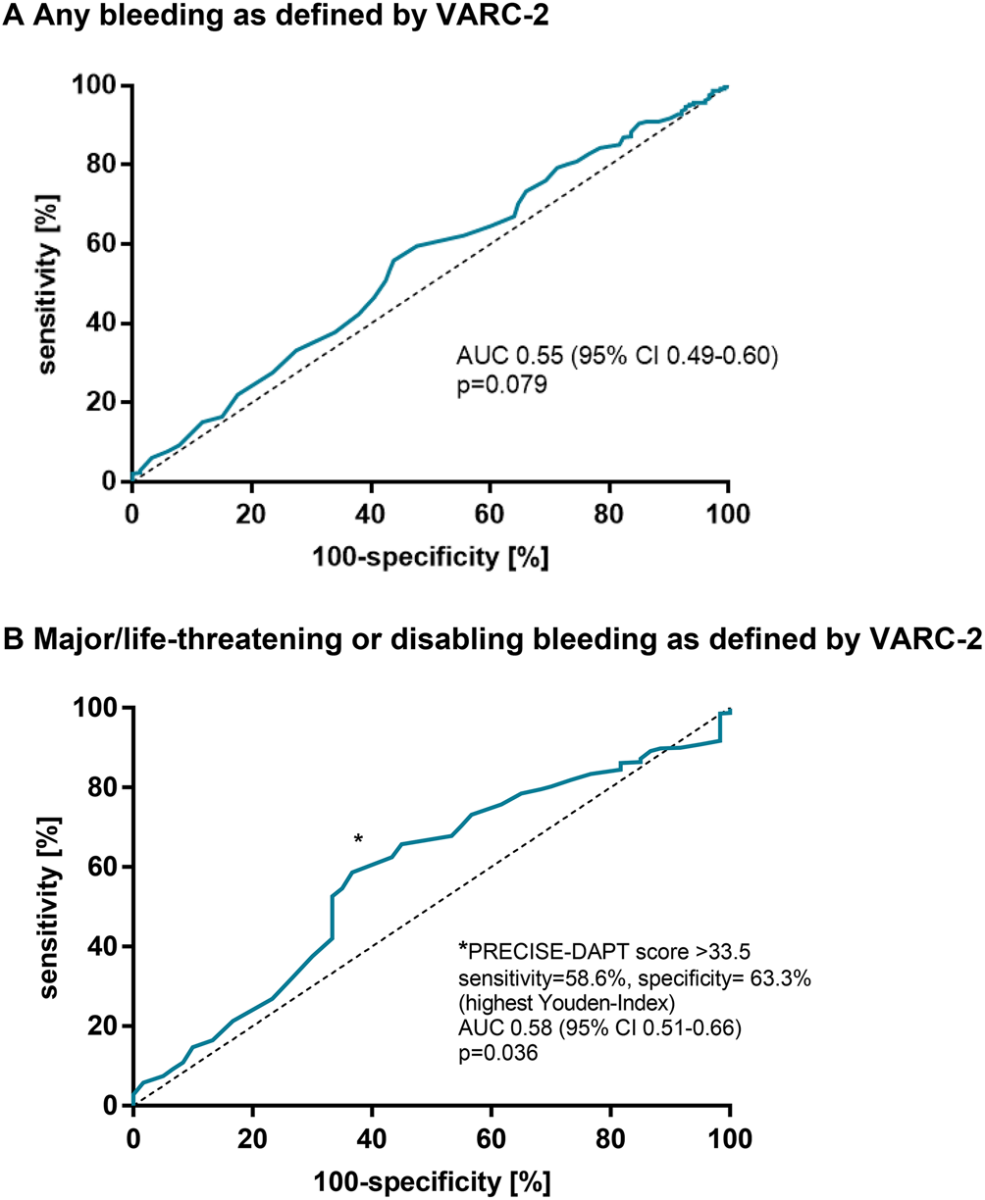
ROC curve analysis evaluating the diagnostic ability of the PRECISE-DAPT score within the DAPT group. **(A)** Based on receiver operating characteristic (ROC) curve analysis, the PRECISE-DAPT score was not significantly associated with the occurrence of any bleeding event. **(B)** In contrast, there was a significant, yet modest association with major/life-threatening or disabling bleeding complications.

Baseline characteristics are presented in Table 1. Patients in the DAPT group were older (median age 82.0 years vs. 81.4 years, p = 0.042) and had significantly higher rates of coronary artery disease, previous PCI, myocardial infarction and previous stroke or TIA (p < 0.001, respectively). In addition, patients with pre-existing DAPT showed a higher prevalence of impaired left ventricular systolic function (32.9% vs. 24.0%, p = 0.032) and peripheral artery disease (13.2% vs. 7.2%, p = 0.026). As a consequence, patients in the SAPT group had a lower Society of Thoracic Surgeons risk score (median 3.2% vs. 4.0%, p = 0.002). Bleeding events are classified in Table 2. Notably, the incidence of periprocedural bleeding and ischemic complications at 30 days as defined by the VARC-2 classification system, did not differ significantly between the two groups. Moreover, the mortality rate at 90 days after TAVR was similar between both groups (Table 3). This finding was consistent in a 1:1 matched analysis which was based on age, gender, BMI, renal function, STS-Score, white blood cell count, platelet count, haemoglobin and the PRECISE-DAPT score (Supplemental Table 1 and 2). In univariable Cox regression analysis, an STS-Score ≥ 4%, major bleeding, life-threatening/disabling bleeding, conversion to open surgery as well as AKIN stage 3 were significantly associated with mortality at 90 days (Table 4). After multivariable analysis, both major bleeding (HR 4.59, 95% CI 1.64–12.83, p = 0.004) and life-threatening/disabling bleeding (HR 8.66, 95% CI 3.31–22.65, p < 0.001) at 30 days were confirmed as significant variables for mortality at 90 days. ROC curve analysis was used to assess the potential diagnostic ability of the PRECISE-DAPT score for VARC-2 bleeding complications among TAVR patients with uninterrupted DAPT (Fig. 2A,B). While a trend, but no statistical significance could be found for any bleeding event (AUC 0.55, 95% CI 0.49–0.60, p = 0.079), there was a significant, yet modest association with major/life-threatening or disabling bleeding complications (AUC 0.58, 95% CI 0.51–0.66, p = 0.036; optimal cut-off for the PRECISE-DAPT score: > 33.5).

**Table 1:** Baseline characteristics of patients undergoing TAVR.

	Total (n = 529)	DAPT (n = 167)	SAPT (n = 362)	P-value
Age (years)	81.7 (78.5–85.6)	82.0 (79.1–86.5)	81.4 (78.2–84.8)	0.042
Female, n (%)	294 (55.6)	83 (49.7)	211 (58.3)	0.065
BMI (kg/m^2^)	26.3 (23.7–29.5)	25.8 (23.6–29.4)	26.3 (23.8–29.8)	0.497
CAD, n (%)	406 (76.7)	166 (99.4)	240 (66.3)	< 0.001
COPD, n (%)	56 (10.6)	19 (11.4)	37 (10.2)	0.688
Diabetes mellitus, n (%)	138 (26.1)	52 (31.1)	86 (23.8)	0.072
Dyslipidaemia, n (%)	276 (52.2)	97 (58.1)	179 (49.4)	0.065
Hypertension, n (%)	470 (88.8)	150 (89.8)	320 (88.4)	0.629
PAD, n (%)	48 (9.1)	22 (13.2)	26 (7.2)	0.026
LVEF ≥ 55%, n (%)	387 (73.2)	112 (67.1)	275 (76.0)	0.032
eGFR (ml/min/1.73 m^2^)	57 (41–72)	56 (44–73)	57 (41–71)	0.669
STS-Score (%)	3.4 (2.3–5.2)	4.0 (2.6–5.7)	3.2 (2.2–5.1)	0.002
Prev. bleeding, n (%)	50 (9.5)	20 (12.0)	30 (8.3)	0.178
Carotid stenosis ≥ 50%	55 (10.4)	20 (12.0)	35 (9.7)	0.419
Prev. stroke/TIA	66 (12.5)	34 (20.4)	32 (8.8)	< 0.001
Prev. CABG, n (%)	69 (13.0)	26 (15.6)	43 (11.9)	0.241
Prev. PCI, n (%)	225 (42.5)	159 (95.2)	66 (18.2)	< 0.001
Prev. MI, n (%)	46 (8.7)	28 (16.8)	18 (5.0)	< 0.001
WBC (× 10^9^/l)	7.2 (6.1–8.6)	7.2 (6.1–8.6)	7.2 (6.0–8.6)	0.654
Platelet count (× 10^9^/l)	230 (191–277)	230 (197–274)	231 (190–277)	0.724
Haemoglobin (g/l)	12.4 ± 1.7	12.2 ± 1.7	12.4 ± 1.7	0.140
**Preprocedural platelet inhibition**				
Aspirin, n (%)	513 (97.0)	167 (100.0)	346 (95.6)	0.004
Clopidogrel, n (%)	174 (32.9)	158 (94.6)	16 (4.4)	< 0.001
Ticagrelor, n (%)	9 (1.7)	9 (5.4)	0 (0.0)	< 0.001

Values are presented as counts (percentages), median (interquartile range) or mean (± SD). A p-value of < 0.05 was considered statistically significant.

*BMI* body mass index, *CABG* coronary artery bypass grafting, *CAD* coronary artery disease, *COPD* chronic obstructive pulmonary disease, *DAPT* dual antiplatelet therapy, *SAPT* single antiplatelet therapy, *eGFR* estimated glomerular filtration rate, *LVEF* left ventricular ejection fraction, *MI* myocardial infarction, *PAD* peripheral artery disease, *PCI* percutaneous coronary intervention, *prev.* previous, *STS-Score* Society of Thoracic Surgeons risk score, *WBC* white blood cell count.

**Table 2:** Description of bleeding events.

	Total (n = 529)	DAPT (n = 167)	SAPT (n = 362)	P-value
Access-site bleeding	112 (21.2)	41 (24.6)	71 (19.6)	0.196
**Non-access site bleeding**				
Pericardial	21 (4.0)	3 (1.8)	17 (5.0)	0.096
Chronic anaemia	19 (3.6)	7 (4.2)	12 (3.3)	0.620
Pulmonary	2 (0.4)	0 (0.0)	2 (0.6)	> 0.999
Intracranial	1 (0.2)	0 (0.0)	1 (0.3)	> 0.999
Gastrointestinal	4 (0.8)	1 (0.6)	3 (0.8)	> 0.999
Urinary	1 (0.2)	0 (0.0)	1 (0.3)	> 0.999
Pacemaker implantation site	1 (0.2)	0 (0.0)	1 (0.3)	> 0.999
Occult	4 (0.8)	2 (1.2)	2 (0.6)	0.594

Values are presented as counts (percentages). Multiple bleeding sites were possible. A p-value of < 0.05 was considered statistically significant.

**Table 3:** Procedural variables and outcomes.

	Total (n = 529)	DAPT (n = 167)		SAPT (n = 362)	P-value
**Procedural variables**					
Self-expanding valve, n (%)	284 (53.7)	94 (56.3)		190 (52.5)	0.415
Balloon-expandable valve, n (%)	245 (46.3)	73 (43.7)		172 (47.5)	0.415
Vascular closure device, n (%)	524 (99.1)	164 (98.2)		360 (99.4)	0.184
**Primary outcomes**					
VARC-2: all bleeding at 30 days, n (%)	153 (28.9)	50 (29.9)		103 (28.5)	0.726
VARC-2: minor bleeding at 30 days, n (%)	93 (17.6)	33 (19.8)		60 (16.6)	0.371
VARC-2: major bleeding at 30 days, n (%)	33 (6.2)	11 (6.6)		22 (6.1)	0.822
VARC-2: life-threatening or disabling bleeding at 30 days, n (%)	27 (5.1)	6 (3.6)		21 (5.8)	0.395
Mortality at 90 days, n (%)	28 (5.3)	6 (3.6)		22 (6.1)	0.298
**Other VARC-2 related outcomes**					
Conversion to open surgery (%)	3 (0.6)	1 (0.6)		2 (0.6)	> 0.999
New pacemaker, n (%)	68 (12.9)	16 (9.6)		52 (14.4)	0.127
Myocardial infarction, n (%)	0 (0.0)	0 (0.0)		0 (0.0)	–
AKIN stage 3, n (%)	4 (0.8)	1 (0.6)		3 (0.8)	> 0.999
Disabling stroke, n (%)	1 (0.2)	0 (0.0)		1 (0.3)	> 0.999
**Postprocedural platelet inhibition**					
Aspirin, n (%)	529 (100.0)	167 (100.0)		362 (100.0)	> 0.999
Clopidogrel, n (%)	520 (98.3)	158 (94.6)		362 (100.0)	< 0.001
Ticagrelor, n (%)	9 (1.7)	9 (5.4)		0 (0.0)	< 0.001
**PRECISE-DAPT**					
Score	31 (25–40)	33 (27–41)		30 (25–39)	0.077
High risk category, n (%)	419 (79.2)	140 (83.8)		279 (77.1)	0.075

Values are presented as counts (percentages) or median (interquartile range). A p-value of < 0.05 was considered statistically significant.

*AKIN* acute kidney injury network, *DAPT* dual antiplatelet therapy, *MACE* major adverse cardiovascular event), *SAPT* single antiplatelet therapy, *VARC-2* Valve Academic Research Consortium-2.

**Table 4:** Cox regression analysis.

Variable	Univariable analysis		Multivariable analysis	
	HR (95% CI)	P-value	HR (95% CI)	P-value
Baseline anemia	2.06 (0.96–4.44)	0.064		
STS-Score ≥ 4%	2.62 (1.22–5.64)	0.014	1.90 (0.85–4.26)	0.117
Major bleeding	3.18 (1.21–8.34)	0.019	4.59 (1.64–12.83)	0.004
Life-threatening/disabling bleeding	10.60 (4.84–23.4)	< 0.001	8.66 (3.31–22.65)	< 0.001
Conversion to open surgery	53.6 (16.1–178.0)	< 0.001	7.68 (0.98–26.89)	0.052
AKIN stage 3	11.7 (2.79–49.40)	< 0.001	9.84 (1.81–32.65)	0.006

Results are presented as adjusted hazard ratios (HR) with 95% confidence intervals (CI). A p-value of < 0.05 was considered statistically significant.

*AKIN* acute kidney injury network, *CAR* CRP/albumin ratio, *STS-Score* Society of Thoracic Surgeons risk score.

## Discussion

In the present study investigating the effect of preprocedural antiplatelet therapy on periprocedural complications in TAVR patients, both pre-existing DAPT and SAPT showed a comparable safety profile in terms of VARC-2 bleeding events at 30 days. In our analysis, any bleeding occurred in 153 patients (28.9%), while major/life-threatening or disabling bleeding was observed in 60 patients (11.3%). Therefore, as a major finding, our study revealed no significant difference between pre-existing DAPT and SAPT with respect to bleeding complications. Based on Cox regression modelling, serious bleeding events (major and life-threatening/disabling bleeding) at 30 days were independently associated with mortality at 90 days after TAVR.

Consistent with our results, no difference in the rates of short-term bleeding complications between preprocedural DAPT and no-DAPT groups was observed in a multi-centre, observational study, which involved a total of 303 TAVR patients^13^. In this study, the incidence of any bleeding was 23.8% with 13.9% of those events classified as major/life-threatening. Compared to our study, the slightly higher rate of major/life-threatening bleeding may be explained by the fact that also non-transfemoral access was used which has been identified as a significant contributor to an increased bleeding risk^14,15^. Similarly, in a small randomized trial, which included 79 patients receiving 18F CoreValve, Ussia et al. found no significant differences between DAPT and SAPT groups regarding life-threatening bleeding during the hospital stay. During 30-day follow-up, the incidence of MACCE (major adverse cardiac and cerebrovascular events), which was defined as a composite of death from any cause, myocardial infarction, major stroke, urgent or emergency conversion to surgery, and life-threatening bleeding, did not differ significantly between the DAPT (13%) and SAPT (15%) group^16^. In another randomized trial including 120 patients, who were randomly assigned to preprocedural DAPT group or aspirin-only group before TAVR, a total of 7 patients (4/60 in the DAPT group and 3/60 in the aspirin-only group) experienced lethal or disabling bleeding during the hospital stay with no difference in the VARC combined 30-day safety endpoint^17^. In a retrospective study evaluating 540 transfemoral TAVR patients, Hioki et al. reported that DAPT before transfemoral TAVR significantly increased the risk of any bleeding during hospitalization compared to SAPT or no antiplatelet therapy^18^. However, these results were derived from the significantly higher incidence of minor bleeding complications in the DAPT group compared to the SAPT or no antiplatelet therapy group. The rates of major and life-threatening bleedings, in consistency with our results, did not differ between the two groups.

In our analysis, the PRECISE-DAPT score showed a significant, yet modest association with major, life-threatening or disabling bleeding at 30 days after TAVR as defined by VARC-2 within the group of uninterrupted DAPT. Thus, the PRECISE-DAPT score may play a role in risk stratification among TAVR recipients who have an indication for pre-existing DAPT.

Regarding postprocedural antiplatelet therapy, the initiation of DAPT after TAVR has been substantially challenged. In the recently published POPular TAVI trial, the difference in bleeding events between SAPT and DAPT groups was driven by all three bleeding severity classifications including minor, major, and life-threatening bleeding, but it was most pronounced in major bleeding^19^. In this trial, most bleeding events occurred in the first month after TAVR. Despite the fact that current guidelines recommend that DAPT should be considered for the first 3–6 months after TAVR, the optimal platelet inhibition regimen remains a matter of ongoing debate. While the POPular TAVI trial has shifted the balance towards SAPT, concerns about thromboembolic complications as well as long-term valve durability may favor a more aggressive treatment strategy^20^. In this context, subclinical leaflet thrombosis has gained growing attention in recent years. Subclinical leaflet thrombosis is characterized by hypo-attenuating leaflet thickening (HALT) on 4-dimensional computed tomography and may progress to hypo-attenuation affection motion (HAM)^21^. HALT is frequently observed in TAVR patients. In the PARTNER-3 trial (Placement of Aortic Transcatheter Valves), the reported incidence of HALT was 28% at 12 months after TAVR^22^. The impact of HALT/HAM on thromboembolic complications and structural valve degeneration is currently investigated in ongoing clinical trials^23^. Importantly, anticoagulation but not antiplatelet therapy is associated with reduced rates of subclinical leaflet thrombosis and has demonstrated to resolve HALT/HAM and restore normal valve function^20,24^. Consequently, a patient-tailored approach balancing both individual bleeding and ischemic risks may become the optimal treatment strategy in the future.

Our study is limited by its single-centre, retrospective design. Thus, a potential selection bias cannot be excluded. In concordance with current guidelines, all patients received DAPT after the procedure. However, this may have significantly contributed to the lack of different bleeding events rates between both groups. Following current clinical practice and expert consensus, computed tomography to detect subclinical leaflet thrombosis/HALT was not routinely performed and thus data on HALT/HAM are not available in our cohort.

## Conclusion

In patients undergoing TAVR who were receiving antiplatelet therapy prior to the procedure, both DAPT and SAPT showed a comparable safety profile regarding periprocedural bleeding complications. If DAPT is indicated, we advocate its uninterrupted continuation in patients undergoing TAVR.

## Supplementary Information

Supplementary Tables.

## Data Availability

The dataset used and analysed in the present study will not be made available due to patient data protection reasons.
