# Supplementary material for: Procedural outcomes in patients with dual versus single antiplatelet therapy prior to transcatheter aortic valve replacement
Source: Sci Rep. 2021 Jul 29;11:15415. doi: 10.1038/s41598-021-94599-2 (PMC8322092; doi:10.1038/s41598-021-94599-2)
Supplement: Supplementary file 1 — Supplementary Tables. [file 41598_2021_94599_MOESM1_ESM.docx]

**Supplemental Table 1.** Baseline characteristics of patients undergoing TAVR (1:1 matched case-control analysis)

|  | Total  (n = 334) | DAPT  (n = 167) | SAPT  (n = 167) | P-value |
| --- | --- | --- | --- | --- |
| Age [years] | 82.2±5.2 | 82.5±5.2 | 82.0±5.1 | 0.359 |
| Female, n [%] | 167 (49.7) | 83 (49.7) | 83 (49.7) | >0.999 |
| BMI [kg/m²] | 26.2 (23.4-29.4) | 25.8 (23.6) | 26.4 (23.2) | 0.939 |
| CAD, n [%] | 277 (82.9) | 166 (99.4) | 111 (66.5) | <0.001 |
| COPD, n [%] | 36 (10.8) | 19 (11.4) | 17 (10.2) | 0.724 |
| Diabetes mellitus, n [%] | 90 (26.9) | 52 (31.1) | 39 (23.4) | 0.110 |
| Dyslipidaemia, n [%] | 166 (49.7) | 97 (58.1) | 69 (41.3) | 0.002 |
| Hypertension, n [%] | 301 (90.1) | 150 (89.8) | 151 (90.4) | 0.855 |
| PAD, n [%] | 34 (10.2) | 22 (13.2) | 12 (7.2) | 0.070 |
| LVEF ≥55%, n [%] | 232 (69.5) | 112 (67.1) | 120 (71.9) | 0.342 |
| eGFR [ml/min/1.73m²] | 56 (43-70) | 56 (44-73) | 56 (42-68) | 0.457 |
| STS-Score [%] | 4.0 (2.6-5.6) | 4.0 (2.6-5.7) | 3.7 (2.5-5.4) | 0.693 |
| Prev. bleeding, n [%] | 36 (10.8) | 20 (12.0) | 16 (9.6) | 0.480 |
| Carotid stenosis ≥50% | 37 (11.1) | 20 (12.0) | 17 (10.2) | 0.601 |
| Prev. stroke/TIA | 49 (14.7) | 34 (20.4) | 15 (9.0) | 0.003 |
| Prev. CABG, n [%] | 46 (13.8) | 26 (15.6) | 20 (12.0) | 0.341 |
| Prev. PCI, n [%] | 37 (11.1) | 28 (16.8) | 9 (5.4) | 0.001 |
| Prev. myocardial infarction,  n [%] | 192 (57.5) | 159 (95.2) | 33 (19.8) | <0.001 |
| WBC [x10^9^/l] | 7.2 (6.0-8.6) | 7.2 (6.1-8.6) | 7.2 (5.9-8.6) | 0.605 |
| Platelet count [x10^9^/l] | 228 (192-273) | 230 (197-274) | 226 (189-273) | 0.342 |
| Haemoglobin [g/l] | 12.2 (11.1-13.3) | 12.2 (11.0-13.2) | 12.2 (11.2-13.5) | 0.735 |
| **Preprocedural platelet inhibition** | |  |  |  |
| Aspirin, n [%] | 326 (97.6) | 167 (100.0) | 159 (95.2) | 0.007 |
| Clopidogrel, n [%] | 166 (49.7) | 158 (94.6) | 8 (4.8) | <0.001 |
| Ticagrelor, n [%] | 9 (2.7) | 9 (5.4) | 0 (0.0) | 0.004 |

*Legend:* BMI, body mass index; CABG, coronary artery bypass grafting; CAD, coronary artery disease; COPD, chronic obstructive pulmonary disease; CVD, cerebrovascular disease; DAPT, dual antiplatelet therapy; SAPT, single antiplatelet therapy; eGFR, estimated glomerular filtration rate; LVEF, left ventricular ejection fraction; MI, myocardial infarction; PAD, peripheral artery disease; PAH, pulmonary arterial hypertension; PCI, percutaneous coronary intervention; prev., previous; STS-Score, Society of Thoracic Surgeons risk score; WBC, white blood cell count. Values are presented as counts (percentages), median (interquartile range) or mean (±SD). 1:1 matching was based on age, gender, BMI, renal function, STS-score, white blood cell count, platelet count, haemoglobin and the PRECISE-DAPT score. A p-value of <0.05 was considered statistically significant.

**Supplemental Table 2.** Procedural variables and outcomes (1:1 matched case-control analysis)

|  | Total  (n = 334) | DAPT  (n = 167) | SAPT  (n = 167) | P-value |
| --- | --- | --- | --- | --- |
| **Procedural variables** |  |  |  |  |
| Self-expanding valve, n [%] | 178 (53.3) | 94 (56.3) | 73 (50.3) | 0.273 |
| Balloon-expandable valve, n [%] | 156 (46.7) | 73 (43.7) | 83 (49.7) | 0.273 |
| Use of vascular closure device, n [%] | 329 (98.5) | 164 (98.2) | 165 (98.8 | >0.999 |
| **Primary outcomes** |  |  |  |  |
| VARC-2: Any bleeding at 30 days, n [%] | 99 (29.6) | 50 (29.9) | 49 (29.3) | 0.905 |
| VARC-2: Minor bleeding at 30 days, n [%] | 61 (18.3) | 33 (19.8) | 28 (16.8) | 0.479 |
| VARC-2: Major bleeding at 30 days, n [%] | 23 (6.9) | 11 (6.6) | 12 (7.2) | 0.829 |
| VARC-2: Life-threatening or disabling ..bleeding at 30 days, n [%] | 15 (4.5) | 6 (3.6) | 9 (5.4) | 0.599 |
| Mortality rate at 90 days, n [%] | 14 (4.2) | 6 (3.6) | 8 (4.8) | 0.786 |
| **Other VARC-2 related outcomes** |  |  |  |  |
| Conversion to open surgery [%] | 2 (0.6) | 1 (0.6) | 1 (0.6) | >0.999 |
| New Pacemaker, n [%] | 37 (11.1) | 16 (9.6) | 21 (12.6) | 0.383 |
| Myocardial infarction, n [%] | 0 (0.0) | 0 (0.0) | 0 (0.0) | - |
| AKIN stage 3, n [%] | 2 (0.6) | 1 (0.6) | 1 (0.6) | >0.999 |
| Disabling stroke, n [%] | 0 (0.0) | 0 (0.0) | 0 (0.0) | - |
| Valve-related dysfunction  (MPG >20 mmHg), n [%] | 0 (0.0) | 0 (0.0) | 0 (0.0) | - |
| **Postprocedural platelet inhibition** |  |  |  |  |
| Aspirin, n [%] | 334 (100.0) | 167 (100.0) | 167 (100.0) | >0.999 |
| Clopidogrel, n [%] | 325 (97.3) | 158 (94.6) | 167 (100.0) | 0.004 |
| Ticagrelor, n [%] | 9 (2.7) | 9 (5.4) | 0 (0.0) | 0.004 |
| **PRECISE-DAPT** |  |  |  |  |
| Score | 33 (26-40) | 33 (27-41) | 32 (26-40) | 0.726 |
| High risk category, n [%] | 281 (84.1) | 140 (83.8) | 140 (84.4) | 0.881 |

*Legend:* AKIN, Acute Kidney Injury Network; DAPT, dual antiplatelet therapy; SAPT, single antiplatelet therapy; VARC-2, Valve Academic Research Consortium-2. Values are presented as counts (percentages) or median (interquartile range). 1:1 matching was based on age, gender, BMI, renal function, STS-score, white blood cell count, platelet count, haemoglobin and the PRECISE-DAPT score. A p-value of <0.05 was considered statistically significant.
